# Exploring the gut microbiome’s role in colorectal cancer: diagnostic and prognostic implications

**DOI:** 10.3389/fimmu.2024.1431747

**Published:** 2024-10-17

**Authors:** Guoming Chen, Qing Ren, Zilan Zhong, Qianfan Li, Zhiqiang Huang, Cheng Zhang, Hongchao Yuan, Zixin Feng, Bonan Chen, Ning Wang, Yibin Feng

**Affiliations:** ^1^ School of Chinese Medicine, Li Ka Shing Faculty of Medicine, The University of Hong Kong, Hong Kong, Hong Kong SAR, China; ^2^ The First Clinical College of Guangzhou University of Chinese Medicine, Guangzhou University of Chinese Medicine, Guangzhou, China; ^3^ Department of Anatomical and Cellular Pathology, State Key Laboratory of Translational Oncology, Sir Y.K. Pao Cancer Center, Prince of Wales Hospital, The Chinese University of Hong Kong, Hong Kong, Hong Kong SAR, China

**Keywords:** colorectal cancer, gut microbiome, disease detection, disease progression, health research

## Abstract

The intricate interplay between the gut microbiome and colorectal cancer (CRC) presents novel avenues for early diagnosis and prognosis, crucial for improving patient outcomes. This comprehensive review synthesizes current findings on the gut microbiome’s contribution to CRC pathogenesis, highlighting its potential as a biomarker for non-invasive CRC screening strategies. We explore the mechanisms through which the microbiome influences CRC, including its roles in inflammation, metabolism, and immune response modulation. Furthermore, we assess the viability of microbial signatures as predictive tools for CRC prognosis, offering insights into personalized treatment approaches. Our analysis underscores the necessity for advanced metagenomic studies to elucidate the complex microbiome-CRC nexus, aiming to refine diagnostic accuracy and prognostic assessment in clinical settings. This review propels forward the understanding of the microbiome’s diagnostic and prognostic capabilities, paving the way for microbiome-based interventions in CRC management.

## 1 Introduction

Colorectal cancer (CRC) is a significant health challenge worldwide and is a leading cause of cancer-related deaths globally (1, 2). According to a study, the link between gut microbiota and CRC seems to be primarily present in the white population, and this connection varies among different races. Certain bacteria are significantly associated with CRC in white women, while the link between Bacillus and CRC is most prominent in black women (3). The development of the CRC condition is influenced by genetic predisposition, lifestyle factors like physical inactivity and smoking, dietary habits such as the consumption of red and processed meats and alcohol, and exposure to various environmental substances (4). Research has shown that chronic psychosocial stress may increase inflammation, disrupt the gut microbiome, and increase the risk of early-onset colorectal cancer (5). All of these factors contribute to the complex nature of the condition. Despite advances in screening and treatment, early detection and accurate prognosis are crucial for improving survival rates. Recent studies have shown that the gut microbiome plays a pivotal role in CRC pathogenesis, indicating its potential for developing non-invasive diagnostic and prognostic tool (6).

The gut microbiome is a complex group of microorganisms that reside in the human gastrointestinal tract. It has been increasingly recognized for its impact on host health and disease development, including colorectal cancer. Studies have shown that an imbalance of gut microbiota, known as microbial dysbiosis, is linked to the initiation, progression, and treatment response of CRC (7). This review aims to explore the current understanding of the gut microbiome’s role in CRC, with a focus on how it can improve diagnostic accuracy and prognostic assessment.

There is mounting evidence to suggest that the gut microbiota plays a crucial role in the initiation, progression, and metastasis of CRC (8–10). In terms of gene count, the human gut microbiota surpasses the human genome, despite the vast array of microorganisms present (11, 12). A healthy gut microbiota is essential for various critical roles, such as energy acquisition, defending against pathogens, shaping the intestinal epithelium, and maintaining immune function (13–16). Dysbiosis of the gut microbiota can disrupt host physiological functions, leading to various diseases (17, 18). The gut microbiota is also influential in several crucial physiological processes, including metabolism, immunity, and the production of nutritional substances (19).

Kostic et al. proposed a mechanism in which Fusobacterium nucleatum increases the concentration of immune cells that infiltrate tumors, leading to the establishment of an inflammatory environment that promotes the progression of CRC (20, 21). Wu et al. reported that enterotoxigenic Bacteroides fragilis (ETBF) secretes toxin BFT, which stimulates the translation and transcription of the proto-oncogene c-Myc, resulting in sustained cell proliferation (22). ETBF induces colitis, activates signaling transducer and activator of transcription 3 (STAT3), and T-helper 17 cell responses in multiple intestinal neoplasia (Min) mice with colon tumors (23). Researchers have identified cyclopropane rings in colibactin secreted by Escherichia coli, which may induce DNA alkylation *in vivo*, potentially contributing to the development of CRC (24). Rohani et al. demonstrated at the genetic level that E. coli K-12 can be considered an important probiotic that may reduce the risk of CRC development (25). There is limited research on the role of bacterial depletion in the development of intestinal tumors due to a lack of appropriate technologies. The absence of certain bacterial strains that are either protective or beneficial may be as crucial as the overgrowth of bacteria associated with tumors (26). The gut microbiota’s diversity, relative abundance, and distribution differ significantly between adenoma patients and healthy individuals (27). CRC patients have alterations in the abundance of bacteria such as Fusobacterium, Enterococcus, and Prevotella, which are associated with mucosal gene expression patterns and may contribute to CRC development. Studies have also shown an increased incidence of CRC in individuals with Salmonella enterica infection. The overall abundance of Enterobacteriaceae in CRC tissues exceeds that in normal tissues by more than 400 times (28).

There are different theories about the relationship between CRC and the gut microbiota. One of these theories is the alpha-bug hypothesis, which suggests that certain bacterial groups can cause cancer and change the bacterial community in a way that supports cancer growth (23, 29–32). Another theory is the CRC gene driver-passenger model, which divides microbiota into driver and passenger bacteria and explains differences in research findings (33, 34).

We analyze how gut microbiota contribute to CRC by examining the findings from recent metagenomic studies. We explore their effects on inflammation, immune modulation, and metabolic processes. Furthermore, we evaluate the potential of emerging microbiome-based biomarkers for CRC screening and prognosis. These biomarkers could complement existing diagnostic methods and facilitate personalized treatment strategies. By summarizing these advancements, we underscore the importance of microbiome research in the quest for better cancer management strategies. This introduction sets the stage for a detailed examination of the gut microbiome’s diagnostic and prognostic potential in CRC.

## 2 Deciphering the gut microbiome: toward early detection and precise prognosis in CRC

### 2.1 Underlying mechanisms: how the gut microbiome influences CRC development

#### 2.1.1 Inflammation and immune

**Figure 1:**
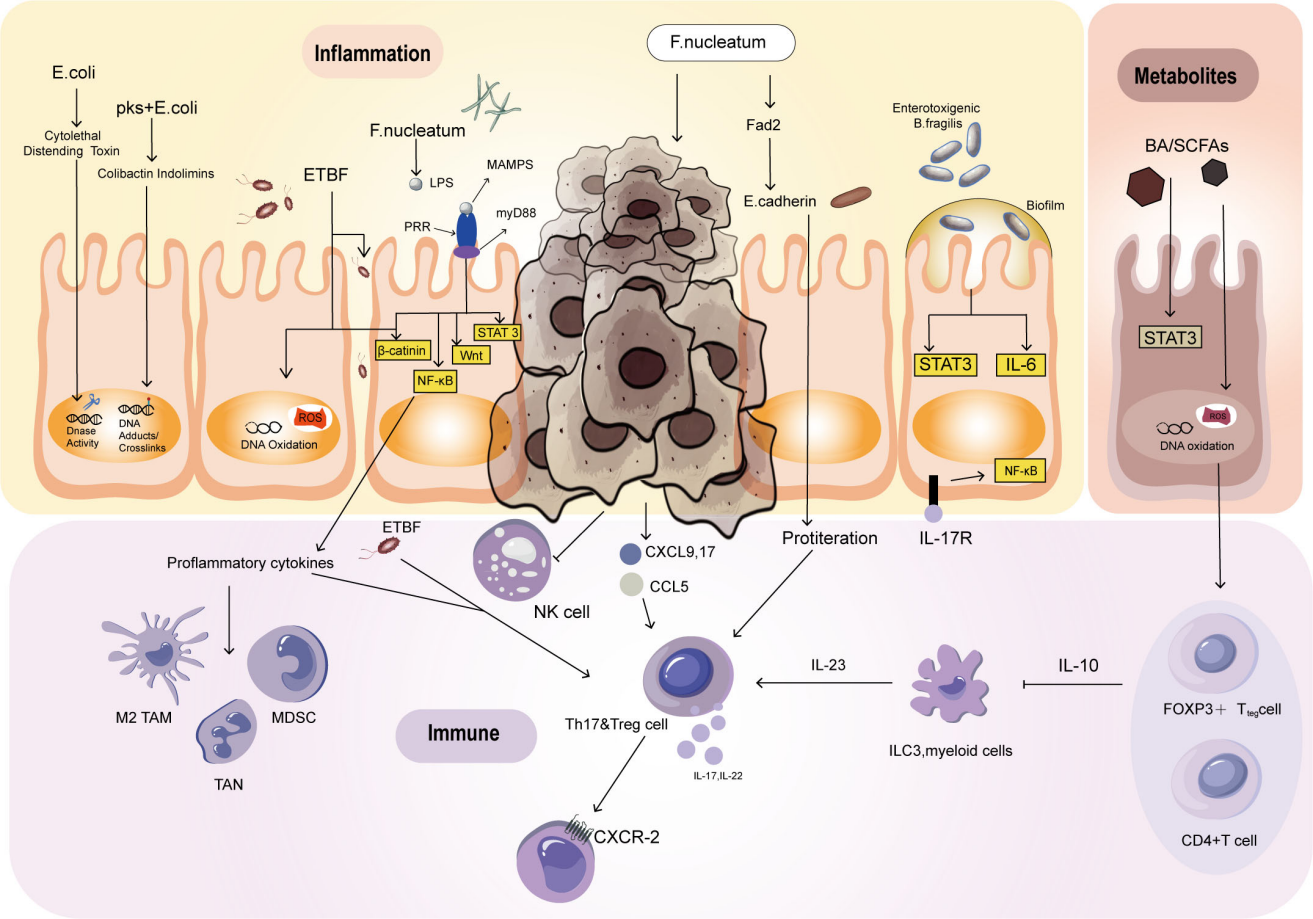
Mechanisms by which the intestinal flora affects the development of CRC. Escherichia coli and ETBF can cause DNA damage. The development of colon tumors is closely associated with the activation of STAT3 and the Th17 response. BA, SCFAs, ETBF, and F. nucleatum can activate STAT3, promoting tumor initiation and growth. However, STAT3 activation alone is not sufficient to trigger colon tumors. Escherichia coli participates in tumor development through Th1 and Th17 immune responses, which induce inflammation. F. nucleatum inhibits NK cells by interacting with T-cell immune receptors. After activating STAT3, some bacteria lead to IL-17-dependent NF-kB activation, resulting in CXCR2 expression and promoting the recruitment of bone marrow cells. SCFAs produced through metabolism promote the differentiation of naive T cells into Th1 cells. BA induces oxidative DNA damage and activates NF-κB, affecting overall immune function.

Colorectal cancer (CRC) and inflammatory bowel disease (IBD) are both characterized by ecological imbalances that can disrupt the mucosal barrier, leading to inflammation and the development of cancer. This ecological imbalance can create a positive feedback loop, which forms the foundation of the inflammation dysplasia cancer sequence (35). It is worth noting that the presence of IBD is often linked to an increased risk of CRC in younger people, but it is not a prerequisite for developing CRC (36–38). Inflammatory reactions can break down the barrier and perpetuate the process of inflammation by causing bacteria to migrate to the intestinal cavity and initiate immunity and inflammation. Inflammatory cells can promote the growth of CRC and induce maldevelopment. Under normal circumstances, epithelial cells reduce oxygen utilization to create an anaerobic environment. However, chronic inflammation leads to an increase in oxygen availability, creating an ecological imbalance that allows pathogenic bacteria such as E. coli to proliferate. These bacteria produce colibactin, a toxin that can destroy DNA and stimulate the growth of tumors. Colibactin induces DNA double-strand breaks by alkylating DNA bases, which triggers replication stress and promotes mutagenesis (39). This direct DNA damage can accumulate over time, ultimately driving tumorigenesis (40). In addition to being a direct DNA-damaging agent, colibactin is linked to inflammation: colibactin-induced DNA damage can activate inflammatory pathways, such as NF-κB, triggering the release of inflammatory cytokines (41). This sets off a feedback loop in which DNA damage triggers inflammation (42), and inflammation further promotes bacterial colonization and DNA damage (43). Over time, chronic inflammation and DNA damage synergistically drive CRC by enhancing cell proliferation and mutagenesis (44), and genomic instability (45). As a result, the inflammatory environment in the gut not only facilitates the colonization of colibactin-producing bacteria but also amplifies DNA damage, further driving cancer development. Moreover, ETBF produces a toxin that triggers inflammatory signaling, promoting the growth of colorectal cancer (**Figure 1**) (35). The toxin known as Bacteroides fragilis toxin (BFT), also referred to as fragilysin (46). BFT is a metalloprotease that specifically targets the E-cadherin protein, which plays a critical role in maintaining tight junctions between epithelial cells (47). The toxin cleaves E-cadherin, thereby disrupting the epithelial barrier and resulting in increased intestinal permeability (48). This disruption triggers an inflammatory response by activating the nuclear factor kappa-light-chain-enhancer of activated B cells (NF-κB) pathway (49). BFT’s activity can lead to diarrhea and inflammation, and it has been linked to the promotion of colorectal cancer due to its role in chronic inflammation and cellular alterations (50).

**Figure 2:**
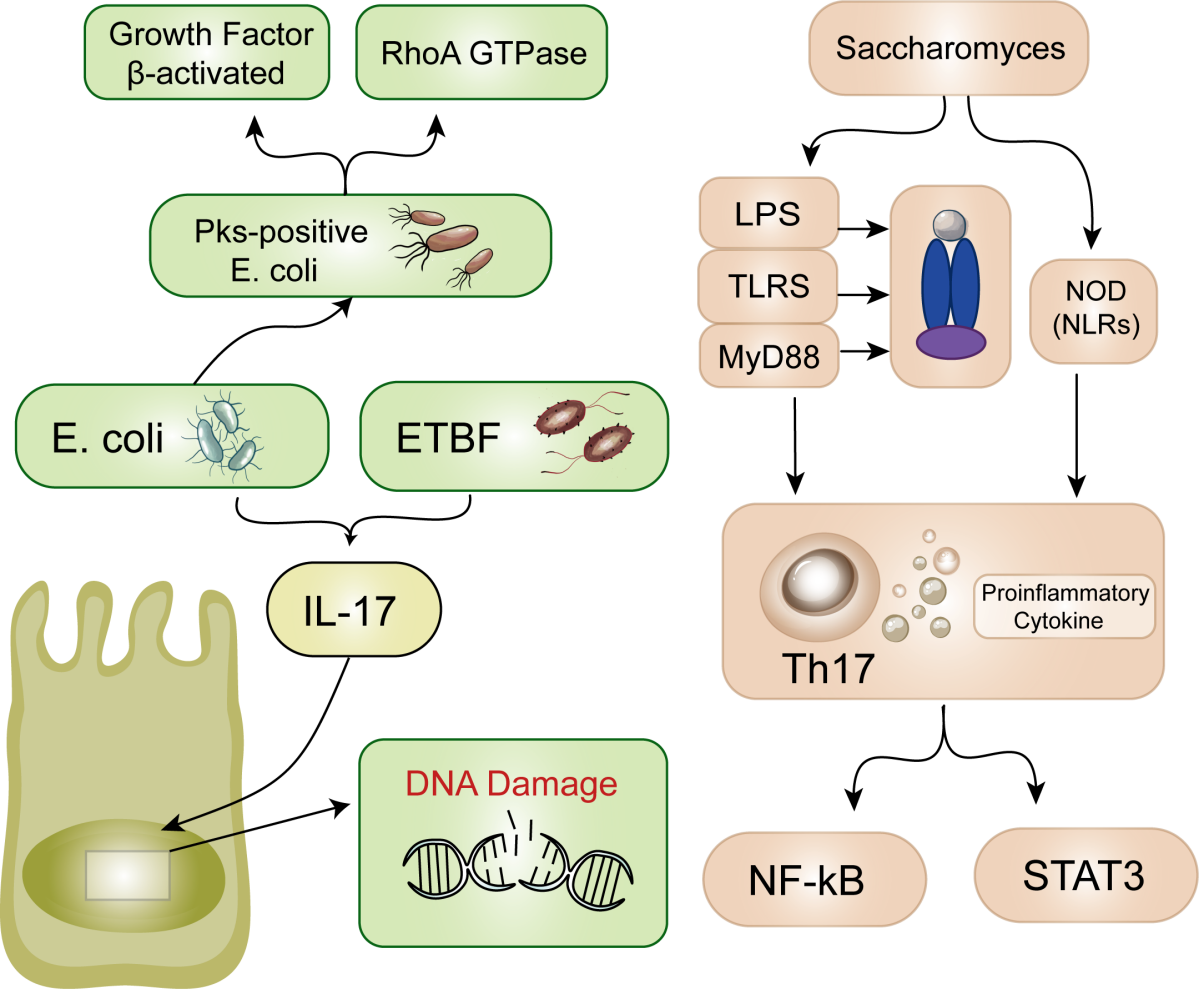
Inflammation. Escherichia coli and ETBF lead to increased generation of IL-17 followed by DNA damage. Additionally, pks-positive Escherichia coli promotes the development of cancer through two pathways. S. cerevisiae induces TLRs and NOD. Thus, TH-17 cells and proinflammatory cytokines are secreted. These receptors act through NF-κB or barrier STAT3.

In patients with IBD, damage to the mucus layer allows bacteria to penetrate the mucosa, resulting in hyperplasia and inflammation (51). Inflammatory damage-induced mucosal disruption allows more bacteria to enter, forming a malignant positive feedback loop of antigen exposure and mucosal injury (52). ETBF is the most common anaerobic bacterium, and its presence is positively correlated with IBD and CRC (53). According to the research of Appunni et al., the cocolonization of toxigenic E. coli and ETBF in mice leads to increased generation of proinflammatory IL-17 followed by DNA damage, potentially accelerating CRC development (**Figure 2**) (54). Currently, E. coli strains containing pks islands express colibactin genes, which have certain effects on the host’s DNA, genes, and chromosomes (**Figure 1**) (55). This may be due to the association of E. coli effector proteins with the DNA mismatch repair system (56). Recent findings have also identified this pathogenic island in E. coli (57). Pks-positive E. coli promotes the development of cancer by activating transforming growth factor β-activated kinase 1 and RhoA GTPase (58). Minute amounts of Saccharomyces cerevisiae, a yeast, induce a Th-17 cell response and proinflammatory cytokine secretion via the activation of signaling pathways mediated by Toll-like receptors (TLRs) and nucleotide-binding oligomerization domain (NOD)-like receptors. These receptors, which act through NF-κB or STAT3, serve as primary sensors for bacterial products and play crucial roles in the prevention of CRC and the inhibition of inflammatory processes (**Figure 2**) (59). According to previous studies, Streptococcus bovis induces the release of inflammatory cytokines (TNF-α、IL-6、IL-1β、IL-8) in CRC cells, creating a pro-inflammatory environment that promotes tumor progression (60). Preliminary clinical trials have demonstrated that ginger consumption leads to a notable decrease in inflammatory markers among participants. While the impact on the overall microbiome appears to be modest, there is a significant reduction in certain microbial taxa, which correlates with the observed anti-inflammatory effects (61). These findings bring hope to those affected by this disease and emphasize the significance of ongoing research on the interplay between microorganisms and cancer cells. Partial inflammation-induced disturbances in intestinal homeostasis directly affect the development of colorectal cancer.

Immune system dysfunction in intestinal epithelial cells plays a crucial role in maintaining the stability of the intestinal microenvironment. Research indicates that infection-induced carcinogenesis is directly associated with endogenous T-cell immune responses.

In a study conducted by Wu et al., it was revealed that the Th17 response plays a significant role in the early stages of carcinogenesis (23). This response is driven by STAT3 activation, which is closely linked to the Th17 response. In mice, ETBF infection induces colitis, strongly promoting colon tumor development. However, the IL-17-mediated signaling pathway and the IL-23 receptor can be blocked using antibodies to inhibit ETBF-induced colitis, colonic hyperplasia, and tumor formation (26). The presence of ETBF can stimulate the recruitment and growth of specific immune cells such as CD4+, CCR6+, IL-17A+, and Th17 cells. This happens through the IL-17 signaling pathway, which can contribute to the initiation and growth of tumors. However, while STAT3 activation is a necessary condition for colon tumor development, it is not enough to trigger colon tumors solely through ETBF (47, 62). IL-17-dependent NF-kB activation leads to CXCR2 expression, promoting the recruitment of myeloid cells and thus facilitating ETBF-mediated colon tumor development (**Figure 1**) (63).

The reduction of ETBF toxin in T lymphocyte utilization of IL-2 promotes the polarization of Th17 lymphocytes and leads to colon tumor development (64, 65). Research on other bacteria, such as Citrobacter rodentium and E. coli, has also revealed their involvement in tumor development through Th1/Th17 immune responses, which induce inflammation (**Figure 1**) (66).

Fusobacterium nucleatum, through interaction with T-cell immune receptors, inhibits the cytotoxicity of NK cells, contributing to the regulation of tumor immunity (**Figure 1**) (67). These studies provide a new perspective for in-depth exploration of the regulatory network between the intestinal microbiome and the immune system.

#### 2.1.2 Metabolism

**Figure 3:**
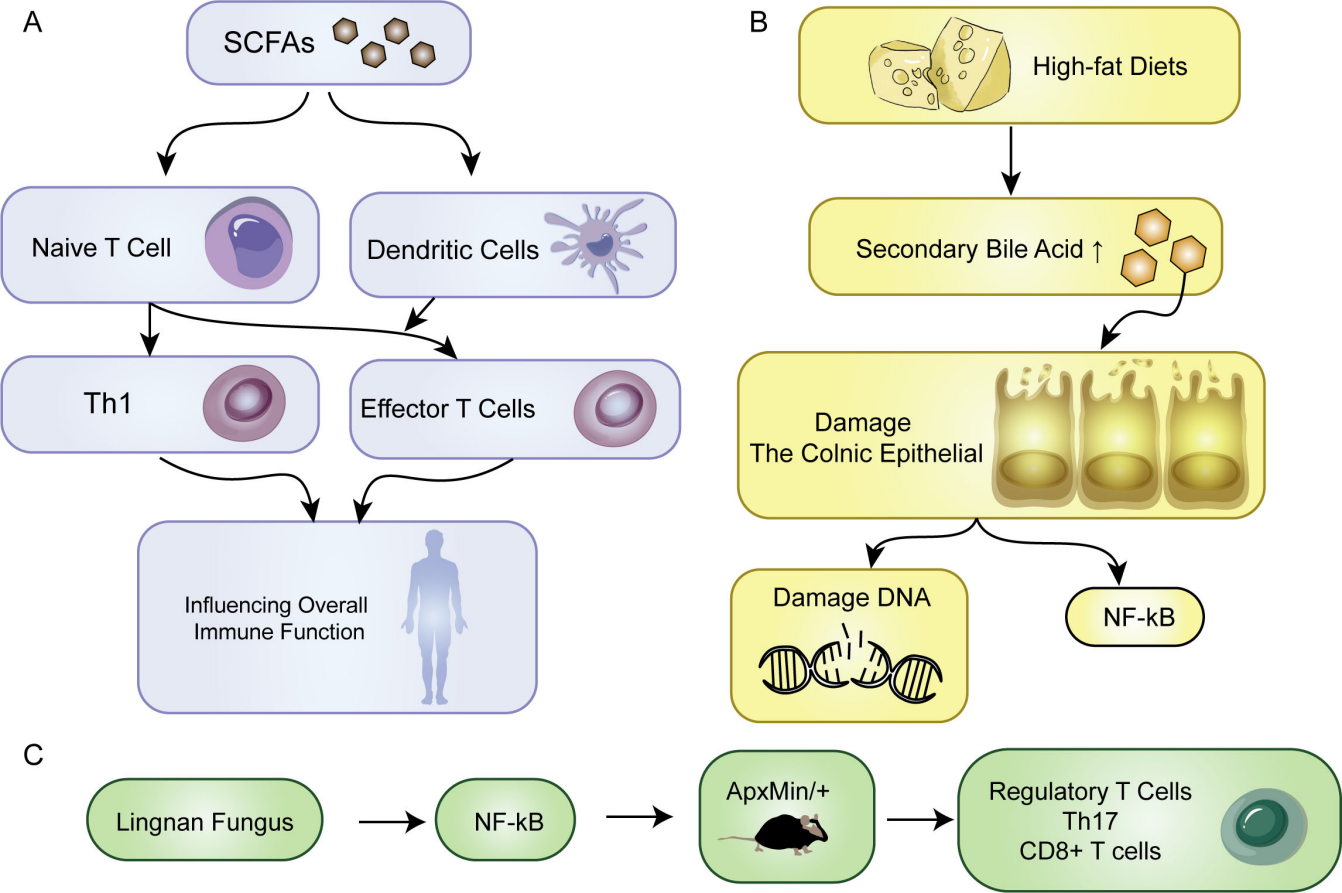
Metabolism. **(A)** SCFAs promote naive T cells to transform into Th1 cells. SCFA-activated dendritic cells direct the maturation of naive T cells into effector T cells, influencing overall immune function. **(B)** The impact of a high-fat diet on the body. **(C)** Effect of Lingnan fungi on the proliferation of tumor bone marrow cells.

The development of tumors is accompanied by changes in metabolic status that affect the microenvironment of the tumor tissue and its surroundings. Both host and dietary components are metabolized together, and the gut microbiota produces various compounds that fulfill their survival requirements while metabolizing different dietary and parasitic components. Diet, in particular, has a significant impact on the metabolic output of the gut microbiota. Bile acids (BAs) and short-chain fatty acids (SCFAs) are important components that play a vital role in the interaction between microorganisms and the host. They are the products of metabolism and can act as protective or harmful agents in the development of CRC (**Figure 1**) (68). The impact of metformin on the gut microbiota has been reported, with some results indicating that metformin increases the abundance of short-chain fatty acid-producing bacteria and mucin-degrading bacteria, thereby promoting the enrichment of beneficial bacteria through metabolic processes (69). The immune system is triggered by the interaction of immune cells with the microbiota in the intestine. This interaction is facilitated by SCFAs produced by various types of bacteria including Firmicutes, Bacteroides, Actinobacteria, Proteobacteria, and Verrucomicrobia (70). SCFAs provide energy to intestinal cells in the host. The activation of immune cells by SCFAs helps in the differentiation of naive T cells into Th1 cells thereby enhancing immune defense. Additionally, dendritic cells that are activated by SCFAs help guide the differentiation of naive T cells into effector T cells thus affecting overall immune function (**Figure 3A**) (71). There are certain bacteria, such as Fusobacterium nucleatum and E. coli, which are related to colorectal cancer. These bacteria impact tumor development and immune responses through different mechanisms. Additionally, the Lingnan fungus activates NF-kB, which in turn induces the proliferation of tumor bone marrow cells in ApcMin/+ mice. Simultaneously, it modulates the frequencies of regulatory T cells, Th-17 cells, and CD8+ T cells (**Figure 3C**) (72). E. coli microbiota stimulation prompts colorectal cancer cells to release chemokines, facilitating the recruitment of T cells into the tumor tissue (73).

In the onset of tumors, metabolites from microbial metabolism play a crucial role. Bacterial bile salt hydrolases catalyze the conversion of primary bile acids into secondary bile acids, including deoxycholic acid and lithocholic acid (74). Individuals consuming high-fat diets commonly exhibit an increase in secondary bile acid in the colon, which is correlated with an increased risk of colon cancer. It contributes to oncogenesis through distinct mechanisms. They directly damage the colonic epithelial barrier, induce oxidative DNA damage, cause genomic instability, and activate NF-κB, collectively facilitating the initiation of tumors (**Figure 3B**). Specific secondary bile acids, such as deoxycholic acid and lithocholic acid, accelerate carcinogen-induced colon cancer, particularly in the presence of a functional mutation in the Apc gene. However, ursodeoxycholic acid, produced by Lactobacillus, plays a protective role in inhibiting CRC development (59). Through comprehensive analyses of the gut microbiota and metabolome, ongoing research continues to unravel the intricate interplay between microbial communities and dietary factors in the CRC environment (75, 76). Polyamines are organic polycations involved in cell proliferation, differentiation, tissue repair, apoptosis, angiogenesis, immune response, signal transduction, and gene expression (77). Elevated levels of polyamine metabolites in biofilms are linked with cell proliferation and CRC. One particular polyamine metabolite, which has been proposed as an early CRC marker, increases in tumors regardless of the presence of biofilm. Compared to healthy tissues, CRC tissues display increased polyamine concentrations, and polyamines promote carcinogenic signaling (78). Current evidence suggests that any polyamine from various sources, such as diet, microbiota, or tissues, can drive tumorigenesis (79). Lysophosphatidic acid (LPA), a promoter of cell proliferation and cell cycle acceleration in CRC cell lines and organoids derived from CRC patients, has emerged as a significant player in CRC pathogenesis. Relevant analysis revealed a close association between infective species and LPA, the metabolic product known to stimulate proliferation (80). Distinct changes are observed in the serum metabolome of CRC patients, particularly in amino acid profiles. The levels of metabolites related to branched-chain amino acids (BCAAs), aromatic amino acids, and phenylalanine typically increase (81). However, evidence indicates that a higher intake of dietary BCAAs may not be associated with an increased risk of CRC but could be related to a lower risk of colorectal cancer (82). Alanine, which was identified as relevant to CRC in one study, has been reported to be a crucial survival signal in certain gastrointestinal tumors (83). Lactobacillus subtilis has the ability to break down tryptophan, thereby producing indole-3 -lactic acid (ILA) that targets ROR γ t. Through this action, it can effectively inhibit Th17 differentiation, reduce IL-17 expression, and potentially prevent the occurrence of colorectal cancer (84).

Metabolic changes in tumor cells can provide valuable information for detecting biomarkers of tumor onset. These changes can more directly reflect the state of tumor cells compared to changes in the genome and proteome. We propose that exploring the relationship between amino acid metabolites and the gut microbiota can reveal distinctive characteristics across various stages of CRC. Studies have suggested that the metabolites produced by the gut microbiota can enter the circulation and regulate distant organs. Therefore, investigating serum metabolites that are closely associated with colorectal cancer holds promise for developing novel diagnostic biomarkers.

Compared to changes in the genome and proteome, metabolic changes can more directly reflect the state of tumor cells. This makes them a promising source for detecting biomarkers of tumor onset. We believe that by studying the relationship between amino acid metabolites and the gut microbiota, we can identify distinct characteristics at different stages of colorectal cancer. Several studies suggest that metabolites produced by the gut microbiota can enter circulation and regulate distant organs. Recent evidence indicates that a relationship exists between sex hormones and the gut microbiome. The gut microbiome’s composition and function, as well as its metabolites, are regulated by sex hormones, while the gut microbiome significantly impacts sex hormone levels. A study has revealed that estrogen may have a protective effect on female CRC (85). Therefore, exploring serum metabolites closely associated with colorectal cancer can hold promising potential for developing novel diagnostic biomarkers. Potential biomarkers for early detection of CRC based on gut microbiome are summarized in **Table 1**.

**Table 1:** Potential biomarkers for early detection of CRC based on gut microbiome.

Marker	Mechanism/Role in CRC	Potential Application	References
Fusobacterium nucleatum	Promotes inflammation, immune evasion, and tumor growth via adhesion to E-cadherin and modulation of the immune system	Early detection, prognosis	(20, 86)
Enterotoxigenic Bacteroides fragilis (ETBF)	Produces Bacteroides fragilis toxin (BFT), disrupting epithelial integrity and inducing chronic inflammation	Biomarker for CRC risk in inflammatory conditions	(87, 88)
Escherichia coli (pks+)	Induces DNA double-strand breaks through colibactin, promoting genomic instability and mutagenesis	Early detection in high-risk individuals	(89, 90)
Bacteroides spp.	Dysbiosis in gut microbiome leads to imbalance in short-chain fatty acids and promotes carcinogenesis	Detection of early dysbiosis linked to CRC	(91)
Short-chain fatty acids (SCFAs)	Anti-inflammatory properties; SCFA dysregulation associated with CRC	Biomarker for microbial dysbiosis and CRC risk	(92, 93)
Lipopolysaccharide (LPS)	Pro-inflammatory component that stimulates immune response; elevated in CRC patients	Biomarker for gut inflammation and CRC development	(94)
Akkermansia muciniphila	Modulates mucin production and maintains gut barrier function; low levels linked to inflammation and CRC development	Prognostic marker for disease progression	(95, 96)
Streptococcus gallolyticus	Associated with CRC through bacterial adhesion, triggering inflammation and promoting carcinogenesis	Marker for CRC risk, especially in CRC-associated bacteremia	(97, 98)
Faecalibacterium prausnitzii	Anti-inflammatory properties through butyrate production, maintaining epithelial barrier integrity	Lower abundance associated with CRC; marker for gut health	(99, 100)

## 3 Microbiome-based diagnostics: revolutionizing CRC detection

**Figure 4:**
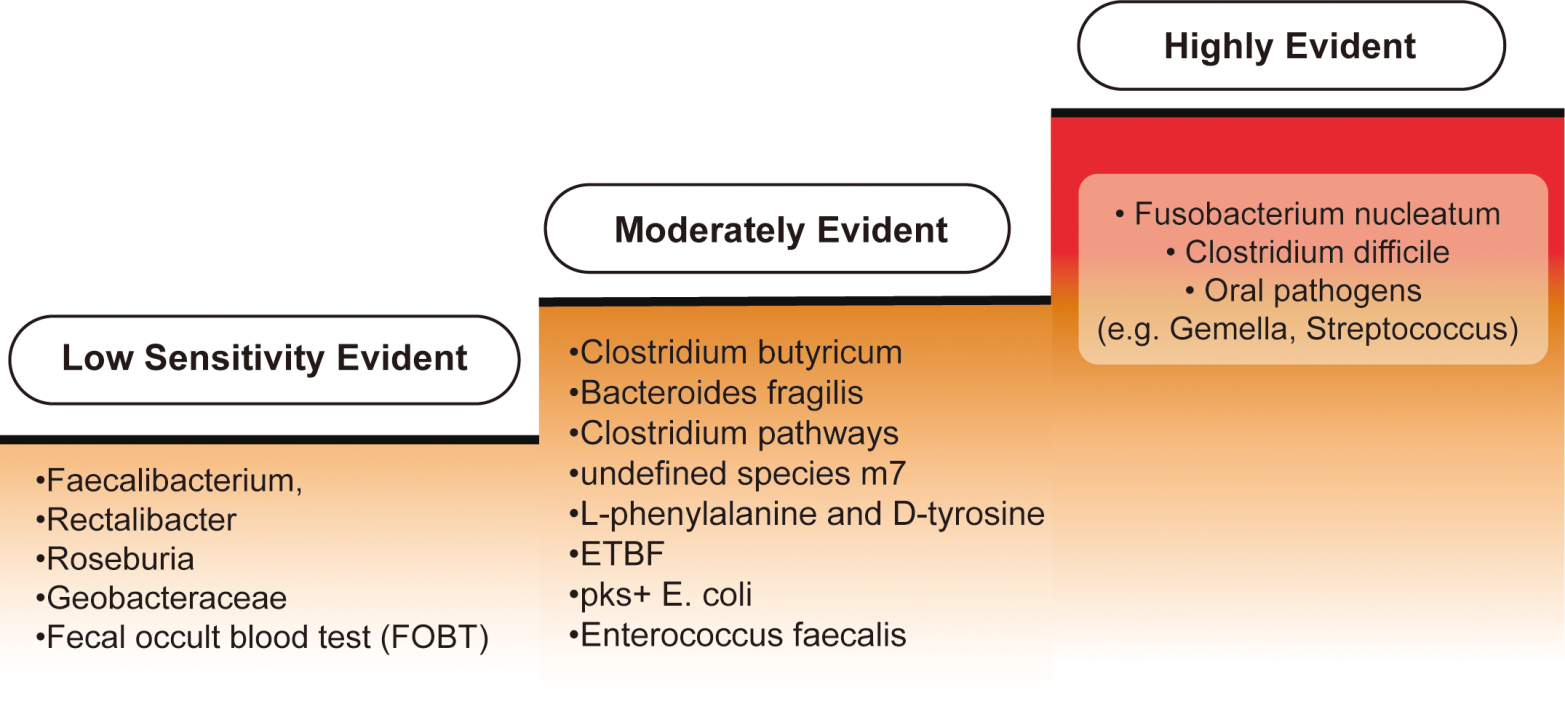
Categorization of biomarkers for colorectal cancer diagnosis. Biomarkers for colorectal cancer (CRC) diagnosis can be categorized into three levels of evidence based on their sensitivity and specificity. The "Highly Evident Biomarkers" group includes Fusobacterium nucleatum, frequently enriched in CRC patients and shown to enhance the sensitivity of the fecal immunochemical test (FIT) when used in conjunction with other diagnostic tools. Additionally, Clostridium difficile has been found to increase in both early- and late-onset CRC cases, suggesting a strong correlation with CRC development. Oral pathogens such as Fusobacterium, Gemella, and Streptococcus are also strongly associated with CRC. In the "Moderately Evident Biomarkers" category, Clostridium butyricum, Bacteroides fragilis, and Clostridium pathways—along with an undefined species, m7—provide moderate detection capabilities, albeit less prominent than the highly evident group. Elevated levels of L-phenylalanine and D-tyrosine are observed in amino acid metabolism specific to early-onset CRC samples. Furthermore, enriched bacteria such as enterotoxigenic Bacteroides fragilis (ETBF), pks+ Escherichia coli, and Enterococcus faecalis are found in CRC patients, though their independent or synergistic roles in CRC development are not fully understood. Lastly, the "Low Sensitivity Biomarkers" category includes reduced levels of Faecalibacterium, Rectalibacter, and Roseburia in late-onset CRC cases, though their absence alone is insufficient for a definitive diagnosis. Geobacteraceae, which exhibits a mutual exclusion relationship with other bacteria, shows weaker evidence for CRC detection. Finally, the fecal occult blood test (FOBT) has limited sensitivity and specificity, often yielding false positives, making it a less reliable biomarker.

According to current research, biomarkers are classified into three categories: highly evident, moderately evident, and biomarkers with low sensitivity for CRC diagnosis, reflecting varying levels of validation. An overview is provided in **Figure 4**.

### 3.1 Microbial biomarkers: pioneering noninvasive screening and diagnosis in CRC

#### 3.1.1 Fecal metagenomic analysis

Studies of gut bacteria associated with adenoma and/or CRC development are summarized in **Table 2**. CRC can be divided into two types: early-onset (EO-CRC) and late-onset (LO-CRC) colorectal cancer. Fecal metagenomic analysis showed that perfluorobutane sulfonic acid (PFOS) continuously accumulated in LO-CRC and BO-CRC samples, indicating potential ecological toxicity (101). In both types of colorectal cancer, L-phenylalanine and D-tyrosine levels increase significantly in amino acid metabolism. However, only in EO-CRC samples, certain amino acids and their microbial derivatives were enriched. In fecal samples, a decrease in butyrate-producing bacteria (Faecalibacterium, Rectalibacter, and Roseburia), a reduction in short-chain fatty acid (SCFA) acetate, and downregulation of GABA biosynthetic genes were observed in LO-CRC (102). Both CRC types showed an increase in Clostridium difficile and Helicobacter pylori, which had a negative correlation with reduced SCFA acetate in LO-CRC (103, 104).

**Table 2:** Studies of gut bacteria associated with adenoma and/or CRC development.

Indicators/Discoveries	Colorectal Cancer Types	Relevant Information/Trends
Perfluorooctane Sulfonic Acid (PFOSA)	LO-CRC	Accumulates continuously in LO-CRC and BO-CRC samples, with potential ecological toxicity (101).
Amino Acid Metabolites	EO-C O-CRC	Levels of L-phenylalanine and D-tyrosine significantly increased in both CRC types, while specific amino acids and their microbial derivatives were enriched only in EO-CRC samples (102).
Butyrate-Producing Bacteria	EO-CRC	Decreased in fecal samples, including fecal Enterobacter, rectal Clostridium, and Roseburia (102).
Short-Chain Fatty Acids (SCFAs) Acetate	LO-CRC	Decreased in LO-CRC, negatively correlated with increased frail spore rods and Helicobacter pylori (103, 104).
GABA Biosynthetic Genes	LO-CRC	Downregulated in LO-CRC (102).
Oral Pathogens	CRC	Elevated levels in the gut microbiota of colorectal adenoma patients, including Clostridium difficile, Moryella, and Lachnoclostridium species (105).
Enriched Oral Pathogens	CRC	Enriched in colorectal cancer patients, including Clostridium difficile, Micrococcus, Streptococcus, etc (106, 107).
Colorectal Resident Bacteria	CRC	Specific colon-resident bacteria enriched in colorectal cancer patients, such as enterotoxigenic frail rod-like bacteria, pks+ Escherichia coli, lithocholic acid chain cocci, etc (108, 109).
Bacterial Combination Testing	CRC	Slightly improved sensitivity in detecting CRC, including Fusobacterium, Bacteroides, Haemophilus, and an undefined species.
Depleted Probiotics	CRC	Includes thermophilic Streptococcus, Streptococcus salivarius, Lactobacillus gallinarum, butyric acid bacillus, and malt-flavored meat rod bacillus, These probiotics engage in competitive or antagonistic interactions with pathogenic microorganisms (110–114).
Fungal Colonization	CRC	Continuously enriched fungi include Rhizopus oryzae, and Cordyceps, while continuously decreased fungi include Aspergillus. Coexistence and mutual exclusion patterns exist between fungi and bacteria (84, 116).
Archaea	CRC	Patient's fecal samples have more halophilic archaea and fewer methane-producing archaea, halophilic bacteria, etc (122).

Apart from colorectal cancer, elevated levels of oral pathogens, including Fusobacterium, Moryella, and Lachnoclostridium, were found in the intestinal microbiota affected by colorectal adenomas, indicating early events of intestinal dysbiosis in colorectal tumor development (105). The enriched oral pathogens in CRC patients include Fusobacterium, Gemella, and Streptococcus (106, 107). Certain colon-resident bacteria, such as ETBF, pks+ E. coli, and Enterococcus faecalis, are enriched in colorectal cancer patients (108, 109). It has been established that certain bacteria are associated with the development of colorectal cancer, but it’s not clear whether they act alone or in combination with other microorganisms to accelerate the disease. A combination of four bacteria found in feces, namely Clostridium butyricum, Bacteroides fragilis, Clostridium pathways, and an undefined species m7, only slightly increased the ability to detect colorectal cancer.

There is a hypothesis that certain beneficial probiotics could be absent in the development of CRC. These probiotics could have mutually exclusive relationships with pathogenic microbes and could also engage in competitive or antagonistic interactions (110–114). Research has aimed to identify factors influencing pathogenic probiotics in the human body, to normalize gut microbiota, improve gastrointestinal barrier function, reduce tumor formation, and inhibit CRC cell proliferation, growth, and metastasis (115). Studies indicate that alterations in the composition of intestinal fungi are associated with colorectal cancer development. Complex relationships between fungi and bacteria, including antagonistic interactions, may contribute to the onset of colorectal cancer. Bacterial differential abundance analysis revealed a significant mutual exclusion relationship only between Geobacteraceae and bacteria. Changes in CRC-specific intra- and interdomain ecological networks suggest coexistence and antagonistic interactions within the bacteria-fungi network, with potential contributions from fungal synergies to CRC development (116). It’s worth noting that the gut microbiome of CRC patients exhibits varying enrichments of specific bacteria based on their body weight. In particular, Actinomyces, Desulfovibrio, and Bacteroides are more prevalent in the gut microbiota of obese CRC patients, while Bacteroides and Prevotella are more abundant in the microbiota of CRC patients with normal body weight (117). Research on archaea indicates an association between the enrichment of halophilic archaea and the reduction of methanogenic archaea with CRC. Studies further revealed mutualistic and antagonistic relationships between archaea enriched in CRC and bacteria enriched or depleted in CRC, collectively promoting CRC (84, 118). Research indicates that bacteria linked to gum disease may spread to the colon, causing microbial imbalance, compromising colonic defense mechanisms, and elevating the levels of harmful metabolites and proteolytic activity, resulting in inflammation and potentially cancerous growth (119). Analysis of mouthwash samples through 16S rRNA gene sequencing indicated that oral pathogenic taxa positively and negatively impact CRC development (120). Several studies, including the one conducted by Flemer et al., have identified particular oral bacteria linked to CRC. They analyzed microbial communities in oral, colonic mucosal tissues, and fecal samples from CRC patients to make these findings. Additionally, after radiotherapy, patients with rectal cancer may experience changes in their oral microbiota due to damage to the intestinal mucosal barrier (121).

#### 3.1.2 Fecal immunochemical test

In CRC screening, the performance of the fecal immunochemical test (FIT) is somewhat limited due to the influence of the critical value of hemoglobin, with a sensitivity ranging from 91% to 71% (123).

#### 3.1.3 Nanogold particles

GNPs have attracted significant interest in disease detection because of their distinctive properties. Colloidal gold immunological analysis technique is a crucial technology in FIT. It serves as a linking scaffold to improve the collective detection sensitivity of DNA and antibodies (124). The colloidal gold approach primarily involves utilizing primary and related secondary antibodies to achieve sensitivities and specificities exceeding 80% for detecting colorectal cancer. Therefore, the colloidal gold method has become one primary approach for colorectal cancer diagnosis (125). Comparing several bacterial species using FIT and fecal occult blood tests revealed that incorporating F. nucleatum screening can enhance the accuracy and sensitivity of FIT. When combined with other bacterial strains, it showed increased sensitivity and specificity. SEPTIN9 combined with FIT improved the sensitivity of CRC detection to 94.4-98%, while maintaining a specificity of 69%. Fecal microbial markers have demonstrated potential in detecting colorectal adenomas, with a combination of several bacteria obtaining an area under the curve (AUC) of 0.90 (126, 127). The determination of differences in BAs and SCFAs in CRC patients provides a direction for further large-scale clinical studies (68).

#### 3.1.4 Fecal occult blood test based on guaia wood resin

There are two methods: the FIT and the fecal occult blood test (FOBT). It can be utilized to detect the presence of blood in the feces. FOBT has benefits including speed, convenience, and noninvasiveness, however its sensitivity and specificity are rather limited. FOBT utilizes tetramethylbenzidine (TMB) as a chromogen, enabling quick findings for at-home testing. However, its drawbacks consist of dietary constraints and a potential for false positive results (128, 129).

In addition to the conventional detection methods mentioned above, we have also summarized some new biomarker methods in **Table 3**.

**Table 3:** Other biomarkers.

Biomarker	Detection Method	CRC diagnosis AUC	Research Findings
GMSM Model	LASSO algorithm selects metabolite features	0.93 (early-stage CRC)	The GMSM model, correlating serum metabolites with colorectal cancer and adenoma, accurately distinguishes patients from healthy individuals. Its performance significantly exceeds that of CEA and FOBT, holding promise as a noninvasive method for colorectal cancer and adenoma detection (130, 131).
Targeted Metabolite Analysis	Orthogonal partial least squares discriminant analysis and ROC analysis	0.969	Metabolomic analysis reveals different enrichment pathways of intestinal metabolites in colorectal cancer and adenoma. Combined analysis effectively distinguishes patient populations. Certain metabolites, such as 9,10-dihydroxy-12-octadecenoic acid and cholesterol esters, can be used to differentiate colorectal cancer from healthy controls. Relevant metabolites correlate with patient survival (132).
Blood cfDNA Detection	Detects tumor cells, organotypic DNA, and donor DNA in tumors, using CRISPR technology	0.96	Engineered bacteria are employed to detect colorectal cancer cells, organotypic DNA, and donor DNA. This approach holds promise in the diagnosis and treatment of colorectal cancer. Clinically occult and radiologically undetectable minimal residual disease (MRD) during surgery is considered a major source of disease recurrence. Compared to the traditional blood marker CEA, preoperative ctDNA exhibits higher sensitivity, allowing more accurate prediction of recurrence risk (133).
Methylation Biomarker MYO1-G	ddPCR and ROC analysis	0.94	The MYO1-G methylation biomarker, detected using ddPCR technology, accurately distinguishes colorectal cancer patients from normal controls. It is also correlated with disease progression and treatment response. Postoperative monitoring reveals a correlation between methylation levels and patient treatment response and survival, providing a new biomarker for colorectal cancer diagnosis and monitoring (134–136).

## 4 Prognostic power of the microbiome: shaping CRC outcomes

The most effective diagnostic techniques for CRC are intrusive and expensive. Developing sensitive, noninvasive, and cost-effective approaches for detecting and predicting the outcome of colorectal cancer is crucial to improve the likelihood of a cure (137).

### 4.1 Survival insights: the microbiome’s prognostic significance in CRC

Mima et al.’s research found that elevated nuclear F. nucleatum levels in colorectal cancer tissues were linked to negative clinical outcomes, such as reduced survival periods and unfavorable prognoses (138). Recent research has sought to validate the hypothesis that greater amounts of F. nucleatum nuclear DNA in tissues may be correlated with adverse clinical outcomes in patients with colorectal cancer. By examining databases of 1,069 CRC patients from two nationwide prospective cohort studies in the United States, it was found that increased amounts of nuclear F. nucleatum nucleoplasmic DNA were associated with increased colorectal cancer-specific mortality rates. Recent research suggests that F. nucleatum may promote the development of colorectal cancer through the expression of the virulence factor FadA. Moreover, this study indicated that tissue-resident F. nucleatum may inhibit T-cell-mediated immune responses, which is correlated with unfavorable clinical outcomes in patients with colorectal cancer. These findings support the notion that high F. nucleatum expression may indicate a more invasive subtype of colorectal cancer. Future research may need to further explore the impact of tissue-resident F. nucleatum on T-cell-based immunotherapy. Overall, nuclear DNA may serve as a biomarker for CRC prognosis, but further validation in other populations is needed. These findings provide insights for developing colorectal cancer prevention and treatment strategies targeting the microbiota (138).

Wang et al.’s study showed that combining regorafenib with toripalimab is safe, effective, and improves survival in colorectal cancer patients. The study indicated that the results of the gut microbiota, particularly the negative correlation between Bacteroides and response and survival rates, provided a combined treatment option for refractory metastatic colorectal cancer patients (139).

This prospective study aimed to explore the role of the gut microbiota in predicting the response to neoadjuvant chemoradiotherapy (nCRT) in locally advanced rectal cancer (LARC) patients. Yuxi Yi et al. constructed a random forest classifier that successfully predicted the nCRT response using 10 microbial biomarkers. These findings suggest that the gut microbiota may serve as a potential biomarker for predicting nCRT response in LARC patients, with clinical significance for their management (140).

While re-establishing the microbiota following intensive antibiotic treatment is a critical step, limited attention has been directed toward understanding the state of health after microbiota cultivation. In the context of clinical trials evaluating microbiota-based therapies, whether employing fecal microbiota transplantation (FMT) or engineered microbial consortia, it is imperative to incorporate comprehensive, long-term studies of the microbiota. Appropriate metrics for such studies may involve monitoring microbial diversity, examining the existence of multidrug-resistant microorganisms, and determining the prevalence of antibiotic-resistant genes. Assessing the carcinogenic potential of the microbiota may be possible one day, and once validated, these indicators should be incorporated (141).

María Antonia Martínez-Sánchez et al. provided the initial study assessing the effect of preoperative nutritional intervention based on high-fiber intake and high levels of polyunsaturated fatty acids (PUFAs) on changes in the intestinal microbiota and their link with postoperative complications, particularly anastomotic leakage, and site infection. The regulation of the microbiota through dietary intervention has been proven in recent explorations of human testees (142–144). In this way, the consumption of a diet that contains a large amount of PUFAs regulates the composition of the gut microbiota (145). It also favors the development of protective bacteria such as Bifidobacterium and Lactobacillus while reducing the presence of pathogenic bacteria such as Pseudomonas (146–148). In summary, they hypothesize that consuming a diet that has a large content of fiber and PUFA before CRC surgery may result in changes in the intestinal microbiota, making it more balanced and potentially reducing postoperative complications.

Lelde Lauka et al.’s systematic review comprehensively analyzed the relationship between gut microbiota and short-term and long-term outcomes after colorectal cancer surgery. This study revealed that specific bacteria, such as F. nucleatum and Co-abundance Groups, may be independent predictors of prognosis and postoperative complications in colorectal cancer patients. However, due to limited data and the influence of confounding factors, more research is needed to further explain these findings. Overall, this research emphasizes the importance of comparing the microbiota composition before and after surgery, providing insights into how the gut microbiota is linked to the underlying mechanisms of colorectal cancer. When interpreting the results, heterogeneity in studies, sample sources, and analysis methods should be considered. Although evidence is limited, this field holds potential clinical importance for improving colorectal cancer surgery outcomes (149).

Although the value of bacterial markers in colorectal cancer diagnosis still requires further research, this direction provides a promising area due to the wide range of microbes and fewer interfering factors. Future study can go deeper into the utilization of microbiota in diagnosing and predicting outcomes of CRC, particularly in the realms of personalized medicine and microbiome intervention treatment. The efforts will reveal the intricate connection between the microbiota and survival rates in colorectal cancer patients, offering more accurate targets for future treatment options.

### 4.2 Association of the gut microbiota with colorectal cancer disease progression

The gut microbiota has a substantial impact on the initiation, growth, and treatment outcomes of CRC (150). Research in this field involves exploring the potential applications of microbial markers in CRC screening and prognosis. Disease recurrence and metastasis are also being explored.

In addition to their potential applications in CRC screening, microbial markers can also serve as prognostic biomarkers for CRC. In colorectal tissue samples, Fusobacterium nucleatum (Fn) is one of the most common microbes, and it is associated with resistance in colorectal cancer (151, 152). Studies suggest that the enrichment of Fn in colon tissues is correlated with poor prognosis in right-sided colon cancer patients, while Fn-negative right-sided colon cancer patients have a prognosis comparable to that of left-sided colon cancer patients (153). This indicates that Fn may undergo stage-specific changes during colorectal cancer recurrence and metastasis, impacting the response to systemic chemotherapy in palliative treatment settings.

### 4.3 Dysbiosis of the gut microbiota and CRC development

CRC is associated with an imbalance in the gut microbiota, with proximal feces reflecting changes in structure and metabolism (154). Analyzing fecal metabolites in CRC patients and comparing them with healthy subjects may identify potential biomarkers related to CRC and explore the impact of metabolites on CRC progression (65). The progression of colorectal tumors along the adenoma-carcinoma sequence disrupts the homeostasis of intestinal metabolites, leading to significant changes in key metabolic pathways, such as the upregulation of cholesterol metabolites and sphingolipids, which are associated with increased fat intake and cholesterol during CRC development (155, 156).

### 4.4 Reprogramming of amino acid and lipid metabolism in CRC development

In the tumor microenvironment, the reprogramming of amino acid and lipid metabolism contributes to the growth of cancer cells and tumor formation. Metabolomic analysis revealed the enrichment of various metabolites, such as 2-aminoethylphosphonic acid salts and amino acid derivatives, in the CRC group, which is crucial for promoting cancer cell proliferation (157, 158). The presence of these metabolites may serve as potential markers for CRC development.

### 4.5 Detection of microbial translocation in blood and its association with CRC prognosis

The detection of microbial translocation in the blood of CRC patients has prognostic significance. Studies have identified key biological associations with CRC through the grouping of OCS, inferring the metabolic potential of OCS, and emphasizing the association between grouping and disease prognosis (159). Changes in the gut microbiota associated with colorectal cancer, demonstrated through microbial markers, show excellent performance in the early detection of CRC in the precancerous stage with high accuracy (160). This study, employing rigorous validation methods, identified biomarkers derived from the microbiota. This discovery instills optimism for the prospect of noninvasive diagnosis of colorectal adenomas and, in the future, may emerge as a promising target for the treatment of colorectal cancer (161).

## 5 Conclusion and future perspectives

In-depth research has been conducted on the gut microbiota of CRC patients. The key role of the gut microbiota in the three carcinogenic processes of CRC has been revealed. First, the continuous proliferation of the gut microbiota during inflammation disrupts the intestinal ecological balance and damages the mucosal barrier, allowing more bacteria to enter and form a vicious positive feedback loop that accelerates the progression of colorectal cancer. Second, certain metabolites of the gut microbiota, such as polyamines and secondary bile acids, participate in carcinogenic signal transduction, leading to tumor formation. Third, the gut microbiota induces the recruitment and proliferation of immune cells by mediating cytokines, which contribute to tumor initiation and growth. Research in this area reveals that there are distinct variations in the prevalence of particular bacterial species, a reduction in microbial diversity, and changes in metabolites in the gut microbiome of patients with colorectal cancer. They also identified biomarker associations between the gut microbiome and CRC. The tight interaction between these bacteria and host epithelial cells impacts the development of CRC.

The gut microbiota once referred to as the forgotten organ, has gradually unveiled its mysterious nature. Studies indicate that the gut microbiome is crucially involved in the pathogenesis of CRC. The occurrence of CRC has been consistently associated with the gut microbiota in numerous clinical studies and experimental models. Robust functional data have reliably demonstrated microbial involvement in the pathways and molecular mechanisms that lead to CRC. Despite achieving preliminary success, a deeper exploration is still needed to determine whether the gut microbiota is a predictor and/or prognostic biomarker for CRC. Conducting thorough longitudinal studies to monitor the alterations in the gut microbiota of colorectal cancer patients over a prolonged period and analyzing these changes in relation to patient outcomes can aid in pinpointing particular compositional aspects of the gut microbiota that may be linked to survival rates, recurrence rates, and other prognostic indicators in colorectal cancer patients. Metabolomics has become an effective method for detecting tumors. Subsequent research can establish a CRC detection model through metabolomic analysis, increasing the probability of diagnosing CRC and predicting favorable outcomes (162). Interventional studies, such as those involving probiotics, prebiotics, and fecal microbiota transplantation, can be conducted to observe their impact on the prognosis of colorectal cancer patients, thereby validating whether the gut microbiota could serve as a potential therapeutic target. Mutual recognition plays a role in identifying potential agonistic and antagonistic interactions, and if a breakthrough is made in this area, it can facilitate the detection of pathogens and subsequent drug research (59).

The utilization of noninvasive biomarkers in current practice, coupled with the repeated improvement in the AUC, highlights ongoing progress in understanding the correlation between the gut microbiota and CRC incidence. Experimental findings employing predictive random forest classifiers underscore a close association between alterations in the gut microbiota and the initiation of carcinogenesis (140).

Microbiome research, confronted by the challenge of limited samples and a multitude of features, innovates logistic regression techniques with penalty methods such as least absolute shrinkage and selection operator (LASSO), ridge, and elastic net methods. In studying tumor microbiota communities, the integration of artificial intelligence (AI) and high-throughput sequencing aims to identify at-risk individuals early, emphasizing significant features such as mutations, methylation, and structural variations.

Unsupervised learning methods, encompassing clustering and dimensionality reduction, aid in feature selection and visualization. Techniques such as random forest (RF), support vector machine (SVM), and penalty regression explore the relationships between microbiota species and CRC features. Time-event machine learning algorithms, including random survival forest (RSF), gradient boosting machine (GBM), Cox-time, and neural multitask logistic regression (N-MTLR), predict CRC-specific survival potential, providing transparent and interpretable models. These models assist clinicians in accurately predicting patient survival rates, formulating personalized treatment plans, and ultimately enhancing patient outcomes (162, 163).

Although the carcinogenic potential of specific bacterial genera, such as Fusobacterium nucleatum, pks-positive E. coli, and Bacteroides fragilis, has been established, the oncogenic properties of other related genera remain unclear. Research confirms the close relationship between CRC and the gut microbiota, emphasizing the need for further exploration of microbial communities in the tumor microenvironment and their impact on CRC. The dynamic interaction between these microbes and host epithelial cells significantly influences CRC development.

During the literature search, it was noted that relevant clinical data are limited. Future studies should focus on multidisciplinary approaches and extensive clinical data analysis to explore the complex link between the gut microbiota and CRC, leading to improved treatment strategies. Challenges in clinical practice, such as individual variations, tumor staging, and cross-species translation, need to be overcome when exploring more accurate diagnostic and prognostic biomarkers.

Therefore, despite promising initial findings, continuous efforts are needed in future preclinical and clinical research to better comprehend the connection between the gut microbiota and CRC. Overall, the study of the gut microbiota provides unprecedented opportunities to explore new diagnostic, therapeutic, and prognostic strategies for colorectal cancer.
